# Advanced platelet-rich fibrin plus gold nanoparticles enhanced the osteogenic capacity of human mesenchymal stem cells

**DOI:** 10.1186/s13104-019-4750-x

**Published:** 2019-11-04

**Authors:** Dara Ghaznavi, Amirreza Babaloo, Adileh Shirmohammadi, Arezoo Rezaie Nezhad Zamani, Mehdi Azizi, Reza Rahbarghazi, Aisan Ghaznavi

**Affiliations:** 1 Dental and Periodontal Research Center, Tabriz University of Medical Sciences, Tabriz, Iran; 2 Department of Periodontics, Dental Faculty, Tabriz University of Medical Sciences, Golgasht Ave, Tabriz, 5166/15731 Iran; 3 Stem Cell Research Center, Tabriz University of Medical Sciences, Tabriz, Iran; 4 Department of Medical Nanotechnology, Faculty of Advanced Medical Sciences, Tabriz University of Medical Sciences, Tabriz, Iran; 5 Applied Cell Sciences Department, Faculty of Advanced Medical Sciences, Tabriz University of Medical Sciences, Tabriz, Iran; 6 Department of Oral and Maxillofacial Radiology, Dental Faculty, Urmia University of Medical Sciences, Urmia, Iran

**Keywords:** PRF, Gold nanoparticles, Mesenchymal stem cells, Osteogenesis, Differentiation

## Abstract

**Objectives:**

There is still insufficient clinical evidence of platelet-rich fibrin beneficial effects on bone regeneration. Gold nanoparticles have been shown to enhance osteogenic differentiation and bone mineralization. The purpose of this study was to investigate the effect of advanced-platelet-rich fibrin modified by gold nanoparticles on the osteoblastic differentiation of human mesenchymal stem cells.

**Results:**

MTT assay revealed 0.0125 mM gold nanoparticles had no cytotoxic effects on stem cells after 7 days. The addition of 0.0125 mM gold nanoparticle to advanced-platelet-rich fibrin clot increased cell viability compared to the non-treated control group (p < 0.05). 7-day incubation of stem cells with advanced-platelet-rich fibrin modified by gold nanoparticles conditioned media was shown to promote alkaline phosphatase activity compared to the control cells and group treated with advanced-platelet-rich fibrin condition media (*p *< 0.05). By using Alizarin Red S staining, red-colored calcium deposits were observed in the group treated with advanced-platelet-rich fibrin and gold nanoparticles conditioned media in comparison with non-treated cells (p < 0.05). Advanced-platelet-rich fibrin conditioned medium was unable to promote calcium deposition compared to the combination of advanced-platelet-rich fibrin and gold nanoparticles (p < 0.05). Adding gold nanoparticles to advanced-platelet-rich fibrin and fibrin and platelet byproducts could be an alternative strategy to improve osteogenic capacity of stem cells.

## Introduction

During the last decades, platelet concentrates and products have been extensively exploited in dentistry and maxillofacial surgery. Among the various types of platelet products, PRF has attracted a lot of attention. To prepare PRF, a small volume of the blood sample was centrifuged with no addition of materials [1]. To date, several biological and regenerative effects have been proposed for preparation of PRF [2]. It has been determined that the release of multiple cytokines and growth factors is likely to stimulate angiogenesis, mitogenesis, and osteogenesis at the target sites while regulates the immune-related response [1, 2]. In spite of the inherent advantages of PRF application in soft tissue regeneration, there is, however, insufficient clinical evidence for bone restoration and healing [3]. Several modifications have been done to yield PRF with a high regenerative outcome [1]. For instance, Kobayashi et al. [4] introduced a modified PRF that was so-called A-PRF^+^. According to this protocol, 10 mL of the patient’s blood is centrifuged at 1300 rpm for 8 min in glass tubes without any anticoagulants.

They declared that reduced centrifugation time and synthesis steps in the preparation of A-PRF^+^ increased PRF cell content [4]. Meanwhile, the release of different growth factors such as TGF-β1, PDGF, EGF, and IGF is greater in A-PRF^+^ compared to other types of platelet concentrates like Advanced PRF and L-PRF [5].

Recently, AuNPs have been applied for the diagnosis and treatment of diseases due to unique biological and optical characteristics and easy synthesis protocol [6]. Gold nanoparticles are capable of promoting osteogenic differentiation and bone mineralization. The addition of AuNPs to the surface of dental implants was found to significantly improve the osteoblastic differentiation and bone formation around the implant [7]. The critical role of AuNPs size and shape has been previously proved in the differentiation of human PDL stem cells into osteoblasts [8]. The idea of adding NPs to the PRF formulation was done to improve PRF regenerative potential in 2018 by Khorshidi et al. [9]. According to their findings, the addition of silver NPs improved the mechanical properties such as tensile strength and stiffness and anti-bacterial features of the L-PRF. This study is a preliminary attempt to assess the effect of A-PRF^+^ enriched with AuNPs on the osteoblastic differentiation of human mesenchymal stem cells.

## Main text

### Materials and methods

#### Experimental procedure

AuNPs nanoparticles were synthesized and characterized by different methods such as DLS, TEM, and ICP-MS as previously described [10]. We then collected blood samples from volunteers to prepare A-PRF^+^. In the next step, A-PRF^+^ was enriched with AuNPs. The potency of CM from A-PRF^+^ and AuNPs + A-PRF^+^ was evaluated on hMSCs to measure osteogenic potential after 7 days. By measuring ALP content and Alizarin Red S staining we monitored the osteogenic capacity of hMSCs after 7 days treated with A-PRF + AuNPs CM (for more details please refer to Additional file 1 and “Materials and methods” section). All procedure of this study was proved by the Ethics Committee of Tabriz University of Medical Sciences (IR.TBZMED.REC.1398.203).

### Results and discussion

#### Synthesis and characterization of AuNPs

**Fig. 1:**
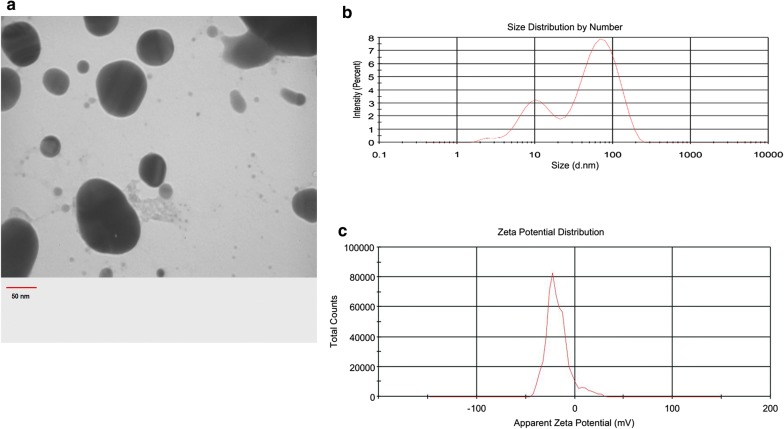
TEM analysis AuNPs. AuNPs are spherical with a mean size of 53 ± 2 nm (**a**) DLS analysis indicated the distribution of AuNP size within the original suspension comparable to TEM imaging (**b**). DLS showed − 19.6 mV zeta potential for synthesized AuNPs (**c**)

Data from TEM analysis revealed spherical particles with diameters 53 ± 2 nm (Fig. 1a). DLS analysis is commonly used to describe the range of nanoparticle size distribution and zeta potential. The polydispersity index, AuNPs diameter, and surface potential were 0.595, 74.7 nm and − 19.5 ± 9.06 mV, respectively (Fig. 1b–c). ICP technique was used to calculate the AuNPs concentration prior to serial dilution preparation. ICP showed an index of 220 mM.

#### A-PRF^+^ and physical properties

**Fig. 2:**
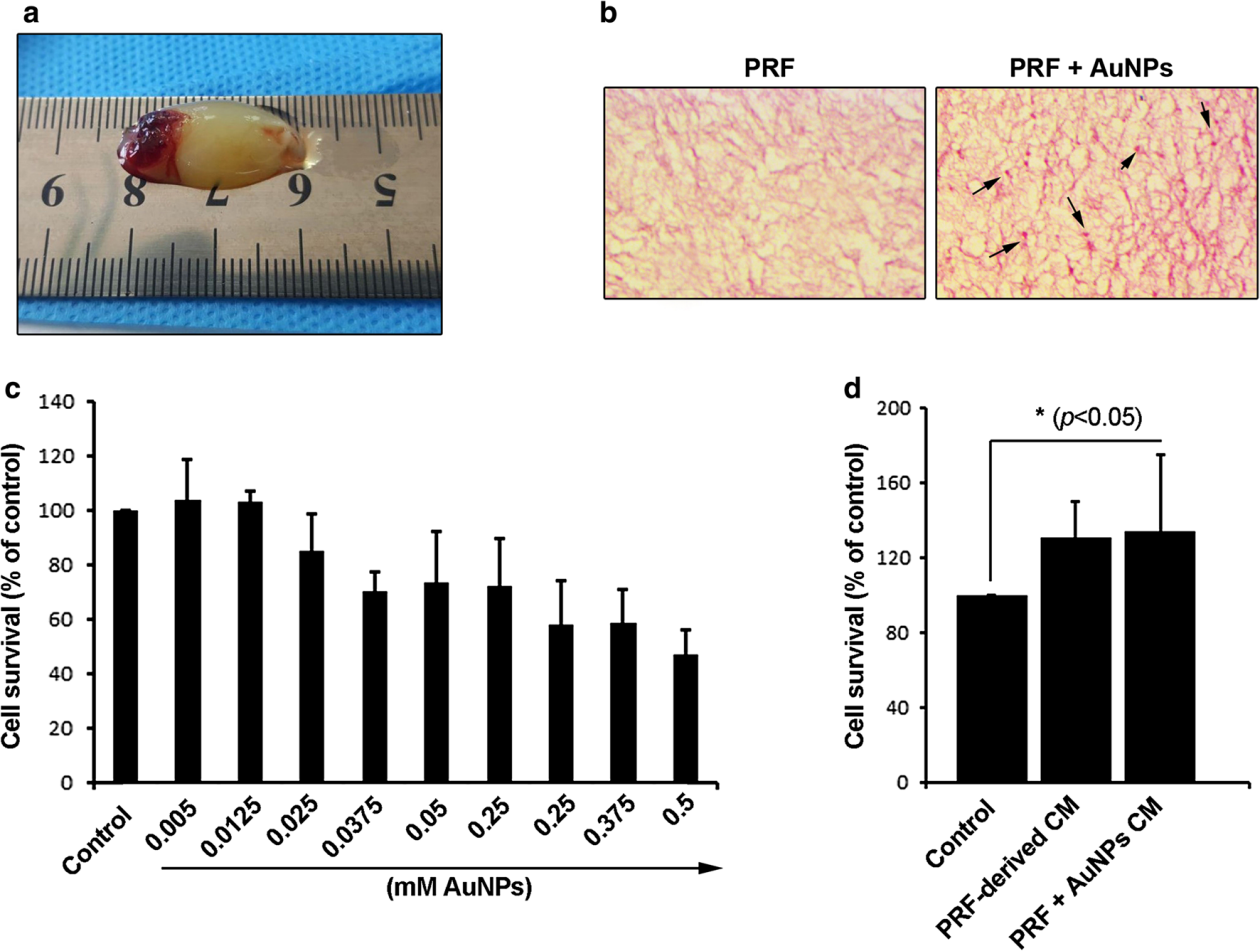
Gross appearance of A-PRF^+^ modified with AuNPs (**a**); bright field imaging of A-PRF^+^ and A-PRF^+^ + AuNPs scaffolds stained H& E. (arrows = AuNPs) (**b**); The analysis of hMSCs viability treated with different concentrations of AuNPs (**c**). MTT analysis of hMSCs treated with CM from A-PRF^+^ and A-PRF^+^ + AuNPs groups over 7 days (**d**) (n = 6). Results show that incubation of hMSCs with CM from A-PRF^+^ plus AuNPs could increase cell survival rate compared to the control group and cells received A-PRF^+^ alone. One-Way ANOVA and post hoc Tukey. *p < 0.05

In the current experiment, we found clots with yellow color and with semisolid stiffness (Fig. 2a). Based on our data, each blood sample (8 mL) yielded 1.5 to 2 mL A-PRF^+^.

#### Histological examination revealed AuNPs trapped by fibrin strands

H&E staining showed the existence of AuNPs in fibrin clot from AuNP-A-PRF^+^, showing the efficiency of our protocol. Bright-field microscopy showed small-sized dense particles trapped between fibrin strands compared to the A-PRF^+^ alone (Fig. 2b).

#### Characterization of A-PRF^+^ conditioned medium

Data from ICP-MS indicated that the concentration of AuNPs in the conditioned medium was 330 ppb.

#### AuNPs decreased the survival rate of hMSCs in a dose-dependent manner

The hMSCs survival rate was calculated after exposure to different concentrations of AuNPs by using MTT assay. Treatment of cells with doses of 0.125 mM and 0.005 mM AuNPs yielded cell viability near to the control levels (Fig. 2c). By exposing hMSCs to AuNPs more than 0.0125 mM, the cell survival rate was decreased a reached minimum levels when cells treated with 0.5 mM AuNPs (Fig. 2c). A reduction of more than 50% was evident in hMSCs from the 0.5 mM AuNPs group. These results demonstrated that AuNPs could modulate the hMSCs viability in a dose-dependent manner. We selected 0.0125 mM for subsequent analyses. In line with our results, Zhang et al. [8] used a very low concentration of AuNPs including 0.1, 1, and 10 μM without cytotoxic effect on PDL progenitor cells. It seems that the application of AuNPs higher concentrations for a short-term period has no detrimental effects on the specific cell type. For instance, Li et al. [11] reported no cytotoxic effect at higher concentrations 0.1, 0.3 and 0.5 mM in hMSCs 3-day incubation.

### Enrichment of A-PRF^+^ clot with AuNPs increased hMSCs viability

The combination of AuNPs with A-PRF^+^ gel was found to increased cell viability rate in a paracrine manner compared to the non-treated control group (*p *< 0.05; Fig. 2d). A non-significant difference was found in the cell survival rate between the control and hMSCs exposed to A-PRF^+^-derived CM (*p *> 0.05). It seems that the addition of AuNPs to A-PRF clot had the potential to significantly increase hMSCs viability (*p *< 0.05; Fig. 2d). It seems that the enrichment of APRF^+^ with AuNPs could accelerate the osteogenic potential of hMSCs in a paracrine manner compared to APRF^+^ alone. Therefore, it seems that the addition of AuNPs may relate to the efficient release of APRF^+^ growth factors and/or stability of the growth factor.

### AuNPs plus A-PRF could accelerate osteogenic differentiation of hMSCs

**Fig. 3:**
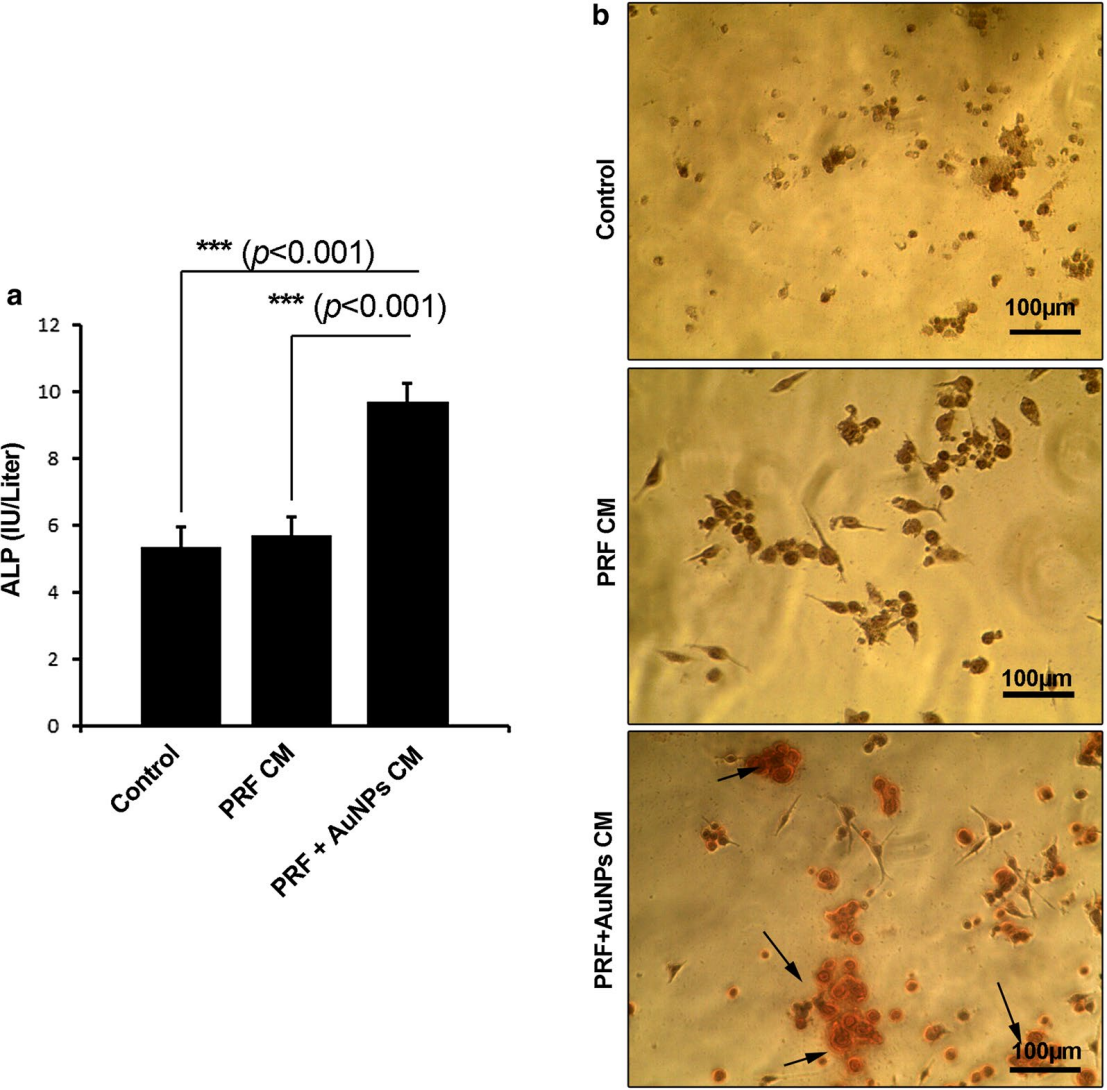
ALP activity of hMSCs treated with CM from A-PRF^+^ and A-PRF^+^ + AuNPs groups after 7 days (**a**; n = 3). Measuring the existence of calcium deposition by Alizarin Red S staining (**b**) treated with CM from A-PRF^+^ and A-PRF^+^ + AuNPs groups after 7 days. Microscopic analysis revealed the potency of A-PRF^+^ + AuNPs CM to promote hMSCs differentiation toward osteoblast-like cells by surrounding calcium deposition indicated by the red-colored matrix. One-Way ANOVA and post hoc Tukey. ***p < 0.001

The osteoblastic differentiation of hMSCs was evaluated using ARS staining and ALP activity (Fig. 3a, b) [12]. Measurement of supernatant ALP in CM showed the ability of hMSCs to release this enzyme following 7-day incubation with AuNPs and A-PRF^+^ mixture (Fig. 3a). Based on the results, the mean concentration of ALP in the supernatant media from the control, A-PRF^+^ and AuNP-A-PRF^+^ groups were 5.3 U/L, 5.7 U/L, and 9.7 U/L, respectively. We found statistically significant difference in released ALP content of the AuNP-A-PRF^+^ group compared to the control and A-PRF^+^ alone $\left( {p_{{{\text{Control}}\;{\text{vs}}.\;{\text{AuNP-A-PRF}}^{ + } }} < 0.001;\;p_{{{\text{A-PRF}}^{ + } \;{\text{vs}} .\;{\text{AuNP-A-PRF}}^{ + } }} \, < \,0.001} \right)$ (Fig. 3a). These data stand for a fact that the conjugation of AuNPs with A-PRF^+^ could enhance osteogenic differentiation by the induction of ALP [13].

By using ARS staining, the existence of extracellular matrix (hydroxylapatite crystals) was evaluated in the control, A-PRF^+^ and AuNP-A-PRF^+^ groups after 7 days (Fig. 3b). Based on microscopic bright field imaging, no calcium precipitation was observed in the control and A-PRF^+^ groups (Fig. 3b). The exposure of hMSCs to CM from AuNP-A-PRF^+^ was found to induce the formation and deposition of red color extracellular matrix indicated with red-colored matrix at the periphery of cells [14, 15].

Microphotographs showed that cells tended to acquire a spindle shape appearance rather than round shape (in the control group) after exposure to secretome from A-PRF^+^ clots with and without AuNPs, showing the inherent activity of A-PRF^+^ to increase cell adherence. Therefore, the exposure of hMSCs to CM from AuNPs + A-PRF^+^ could trigger the osteogenic capacity of hMSCs evident with ALP induction and hydroxyapatite deposition. In contrast, Dohan et al. [16] showed that PRF per se had a positive and dose-dependent effect on the osteoblastic differentiation of hMSCs. Nugraha and co-workers [17] concluded that PRF had the potential to induce the expression of bone ALP and osteocalcin in gingival MSCs. The differences between our results and previous data could be correlated with the incubation time period and type of cells. Li et al. [11] claimed that spherical nanoparticles with a diameter of 40 and 70 nm, especially 70-nm size rod-shaped particles significantly enhanced the genes expression osteogenic lineage.

Cell survival analysis showed that concentrations higher than 0.0125 mM AuNPs could contribute to hMSCs toxicity during the 7-day incubation period. In line with our results, Zhang et al. [8] used a very low concentration of AuNPs including 0.1, 1, and 10 μM without cytotoxic effect on PDL progenitor cells. It seems that the application of AuNPs higher concentrations for a short-term period has no detrimental effects on the specific cell type. For instance, Li et al. [11] reported no cytotoxic effect at higher concentrations 0.1, 0.3 and 0.5 mM in hMSCs 3-day incubation. According to the present study, enrichment of APRF^+^ clots with 0.0125 AuNPs promoted hMSCs survival. One could hypothesize that the combination of AuNPs with APRF^+^ could accelerate hMSCs in a paracrine manner compared to APRF^+^ alone. Therefore, it seems that the addition of AuNPs may relate to the efficient release of APRF^+^ growth factors and/or stability of the growth factor. It should not be forgotten that the application of 0.01 25 mM AuNPs alone could promote hMSCs proliferation and survival. We also applied the ICP-MS test to confirm the release of AuNPs after addition to APRF^+^ clots. This assay confirmed the successful release of AuNPs to the culture supernatant and found to reach 330 ppb. Commensurate with these findings, the current experiment proved the therapeutic paracrine activity of Au-APRF^+^ to modulate hMSCs survival rate. To analyze osteogenic property of AuNPs-APRF^+^, we also monitored the content of released ALP after 7-day incubation with AuNPs-APRF^+^ conditioned medium. The levels of ALP produced by hMSCs exposed A-PRF^+^-derived CM were significantly higher than that of ALP in the control and A-PRF^+^ group. Based on our data, AuNPs-free A-PRF^+^ clot was unable to promote osteogenic differentiation of hMSCs. In contrast, Dohan et al. [16] showed that PRF per se had a positive and dose-dependent effect on the osteoblastic differentiation of hMSCs. Nugraha and co-workers [17] concluded that PRF had the potential to induce the expression of bone ALP and osteocalcin in gingival MSCs. The differences between our results and previous data could be correlated with the incubation time period and type of cells. Under ARS staining, calcium deposition was also prominently visible in the hMSCs treated with AuNP-A-PRF^+^ CM. Along with our results, Li et al. [11] claimed that spherical nanoparticles with a diameter of 40 and 70 nm, especially 70-nm size rod-shaped particles significantly enhanced the genes expression osteogenic lineage. Zhang et al. [8] showed that AuNPs with a diameter of 13 and 45 nm induced osteogenic properties in PDL progenitor cells. Interestingly, the study of PRF exudates on PDL cells showed increased proliferation and osteoblastic differentiation rate [18]. These data stand for a fact that the extraction of PRF could also be an alternative modality for the induction of cell differentiation [19, 20]. However, the effect of the fibrinous substrate must not be neglected in favor of stem cell differentiation toward specific lineages.

## Limitations

There are some limitations related to the current experiment. It seems logical that future investigations monitor prolonged incubation of stem cells with AuNP-A-PRF^+^ CM and the application of different stem cell types will be beneficial to precisely conclude the osteogenic potential of AuNP-A-PRF^+^ CM. We also suggest future experiments to investigate the stability and resistance of growth factors and cytokines trapped in fibrin clots and AuNPs role in the dynamic of these factors.

## Supplementary information

**Additional file 1.** Full description of the methods performed in the present study. It includes the protocol of AuNPs synthesis, PRP preparation, and human stem cells culture, preparation of A-PRF conditioned media, MTT survival assay, and osteogenic analyses. The statistical analysis section is presented in the e-file but not in the article file.

## Data Availability

Data will be available upon reasonable request from the corresponding author.

## Abbreviations

MTT: 3-(4,5-dimethylthiazol-2-yl)-2, 5-diphenyl tetrazolium bromide
ALP: alkaline phosphatase
CM: condition media
DMSO: dimethyl sulfoxide
DMEM/LG: Dulbecco’s modified Eagle’s medium/low glucose
DLS: dynamic light scattering
EGF: epidermal growth factor
AuNPs: gold nanoparticles
FBS: heat-inactivated fetal bovine serum
hMSCs: human amniotic mesenchymal stem cells
ICP-MS: inductively coupled plasma mass spectrometry
IGF-1: insulin-like growth factor-1
L-PRF: leukocyte- and platelet-rich fibrin
PDL: periodontal ligament
PDGF: platelet-derived growth factor
PRF: platelet-rich fibrin
A-PRF^+^: advanced PRF+
TGF-β1: transforming growth factor-beta 1
TEM: transmission electron microscopy
