# Supplementary material for: Advanced platelet-rich fibrin plus gold nanoparticles enhanced the osteogenic capacity of human mesenchymal stem cells
Source: BMC Res Notes. 2019 Nov 4;12:721. doi: 10.1186/s13104-019-4750-x (PMC6827227; doi:10.1186/s13104-019-4750-x)
Supplement: Supplementary file 1 — Additional file 1. Full description of the methods performed in the present study. It includes the protocol of AuNPs synthesis, PRP preparation, and human stem cells culture, preparation of A-PRF conditioned media, MTT survival assay, and osteogenic analyses. The statistical analysis section is presented in the e-file but not in the article file. [file 13104_2019_4750_MOESM1_ESM.docx]

1. **Materials and methods**

***2.1. AuNPs synthesis***

0.5 ml HAuCl_4_ solution (1% w/v) was added to 50 mL of D.W and heated until boiling. Thereafter, 1.6 ml sodium citrate solution (1% w/v) was added to the boiling solution quickly with vigorous stirring. Following color change, the mixture solution was kept boiling for another 15 minutes and allowed to cool down at room temperature while stirring for overnight. Then, 13 nm AuNPs were obtained, and the nanoparticles were kept as the seed solution to synthesize larger gold nanoparticles measuring 50 nm. For this propose, 2.5 ml solution containing 13 nm-sized AuNPs was mixed with 2.5 ml HAuCl_4_ solution (10 mmol/L) and mixture was diluted by adding 150 m D.W. 100 ml ascorbic acid solution (0.4 mmol/L) was added to the above stirring solution. Finally, the 50 nm Au NPs in purple red were obtained. The AuNPs were characterized by different methods such as DLS, TEM and ICP-MS as previously described [10].

***2.2. Blood sampling and PRF Preparation***

In the current experiment, blood samples were collected from healthy volunteers. All procedure of this study was proved by the Ethics Committee of Tabriz University of Medical Sciences (IR.TBZMED.REC.1398.203). To decrease variation related to blood samples, each person was asked to donate 16 ml blood for the preparation of A-PRF^+^ and A-PRF^+^ plus AuNPs. In the next step, blood samples with no anticoagulants were transferred into glass-coated vacuum tubes containing 0.1 ml of 1 mM AuNPs solution. The tubes were shaken gently to yield a transparent solution and centrifuged at 1300 rpm for 8 minutes. After the completion of centrifugation, three separate layers were observed from the top to bottom of the tube including platelet-poor plasma, PRF, and red blood cells. Tubes were allocated under sterile laminar flow hood and PRF mass were separated using a sterile cotton plier and kept at -20°C until use.

***2.3. Cell culture and expansion***

hMSCs were obtained from the Stem Cell Research Center, Tabriz University of Medical Sciences (Tabriz, Iran). hMSCs were re-suspended in DMEM/LG medium (Gibco) with 10% FBS and cultured at 37°C with 5% CO_2_ under humidified atmosphere. The medium was changed every 3-4 days. Cells at passages 3-4 were used in the current experiment.

***2.4. MTT assay***

To assess the effect of AuNPs on hMSCs, cells were exposed to various concentrations of AuNPs ranging from 0.005, 0.0125, 0.025, 0.0375, 0.05, 0.125, 0.25, 0.375, 0.5 and 1 mM. To this end, 2.5 x 10^4^ cells were re-suspended in DMEM/LG with 1% FBS containing AuNPs and plated in each well of 96-well plates (SPL). After 7 days, we added 50 µl MTT solution (dilution: 5 mg/ml; Cat no: M2128) and kept for three hours at 37˚C. Thereafter, DMSO (Cat no: 67-68-5) was poured to each well to dissolve formazan crystals and generate blue-to-purple appearance. Finally, the final absorbance of each well was read at 570 nm by using a microplate reader (BioTek). This assay was done in octuplicate. Based on data from MTT assay, we found that 0.0125 mM AuNPs had a maximum protective effect on hMSCs after 7 days and selected for subsequent analyses.

***2.5. Preparing PRF-derived conditioned medium***

To evaluate the paracrine activity PRF containing AuNPs on osteogenic differentiation of hMSCs, we prepared PRF-derived conditioned medium. PRF masses were incubated in 8 ml DMEM/LG for 72 hours. The medium was then collected, centrifuged for 10 minutes at 1200 rpm to exclude debris and sterilized by using 0.2 µm-microfilters (TPP; Switzerland).

***2.6. Measuring released AuNP to conditioned medium***

The ability of PRF mass to release AuNPs was analyzed by using ICP-MS technique. After the collection of conditioned medium, 100 μl of the medium was mixed with 1.9 ml of Aqua regia (HNO_3_+ 3HCl) and heated to 180˚C for 10 minutes. After 24 hours, samples were diluted with 10 ml solution containing 1% HNO_3_ and 2% HCl and analyzed by ICP-MS technique.

***2.7. Evaluation of released AuNPs on hMSCs***

The possible cytotoxic effect of A-PRF harboring AuNPs on hMSCs was investigated by using MTT assay. As above-mentioned, hMSCs were plated at an initial density of 2.5 x 10^4^ cells per well of 96-well plates. After 24 hours, cells were allocated into three different groups as follows; Control cells that received DMDM/LG with 2% FBS; Cells were given A-PRF-derived conditioned medium enriched with 2% FBS, and cells treated with conditioned medium from AuNP enriched A-PRF containing 2% FBS. Cells were maintained for 7 days and the survival rate was studied by using MTT assay as above-mentioned.

***2.8. Osteogenic differentiation***

***2.8.1. Alkaline phosphatase activity***

ALP production was measured with the alkaline phosphatase assay kit and by the instructions of the manufacturer. First, hMSCs were cultured in 24-well plates containing 500 μl of DMEM/LG with 10% FBS at 37°C and 5% CO_2_. After 24 hours, the media were replaced with CM from A-PRF and A-PRF+ AuNPs and maintained for 7 days. The media were replenished every 3-4 days. After completion of the incubation period, supernatants were collected and centrifuged for 10 minutes at 2500***g***. The content of ALP was calculated by using p-nitrophenyl phosphate method as previously described [19].

***2.8.2. Alizarin Red S staining***

To detect extracellular calcium deposition, hMSCs were stained with Alizarin Red S solution after treatment with A-PRF and A-PRF+ AuNPs conditioned media. In brief, 7 days after incubation with condition media, hMSCs were washed with PBS twice and fixed with 100% methanol (Merck) for 10 minutes and stained with 0.1% Alizarin Red S solution for 30 minutes. In the next step, cells were washed with PBS twice (each for 10 minutes) and examined under a light microscope. For semi-quantitative evaluation, the stained cells were first dried in the air before being washed with 5% HCl for 20 minutes at room temperature. The contents of wells were then transferred to a 96-well plate, and the absorbance of the 405 nm wavelength was measured and normalized to the number of cells.

***2.9. Statistical analysis***

Data are expressed as mean ± SD. Data were statistically compared with each other by One-Way ANOVA analysis and Tukey post-hoc test using SPSS17 software. P<0.05 was considered statistically significant.
